# Predictors of Postoperative Atrial Fibrillation after Coronary Artery Bypass Graft Surgery

**Published:** 2008-04-01

**Authors:** Majid Haghjoo, Hossein Basiri, Mehrasa Salek, Mohammad Ali Sadr-Ameli, Faranak Kargar, Kamal Raissi, Gholamreza Omrani, Mohammad Bagher Tabatabaie, Hassan Mirmohammad Sadeghi, Ali Sadeghpour Tabaie, Ramin Baghaie

**Affiliations:** 1 Department of Pacemaker and Electrophysiology, Rajaie Cardiovascular Medical and Research Center, Iran University of Medical Sciences, Tehran, Iran; 2 Department of Cardiology, Rajaie Cardiovascular Medical and Research Center, Iran University of Medical Sciences, Tehran, Iran; 3 Department of Cardiovascular Surgery, Rajaie Cardiovascular Medical and Research Center, Iran University of Medical Sciences, Tehran, Iran

**Keywords:** atrial fibrillation, predictor, coronary artery bypass graft

## Abstract

**Objectives:**

The present study was aimed to identify the preoperative, intraoperative, and postoperative predictors of AF in a pure cohort of the patients with coronary artery disease who underwent CABG surgery.

**Methods:**

Between November 2005 and May 2006, 302 consecutive patients were included in this prospective study. All the relevant clinical, electrocardiographic, echocardiographic, and laboratory data were gathered in the included patients and they were also monitored for development of post-CABG AF.

**Results:**

Postoperative AF occurred in 46 (15%) of patients. By univariate analysis, older age, P-wave abnormality in ECG, presence of mitral regurgitation, larger left atrium (LA), left main coronary artery involvement, failure to graft right coronary artery (RCA), and adrenergic use in ICU were significantly associated with occurrence of post-CABG AF (all P< 0.05). However, in the logistic regression model, age (OR: 1.067, 95%CI: 1.02-1.116, P=0.005), LA dimension (OR: 1.102, 95%CI: 1.017-1.1936, P=0.017), P-wave morphology (OR: 12.07, 95%CI: 3.35-48.22, P=0.0001), failure  to graft RCA (OR: 3.57, 95%CI: 1.20-10.64, P=0.022), and postoperative adrenergic use (OR: 0.35, 95%CI: 0.13-0.93, P=0.036) remained independently predictive of postoperative AF.

**Conclusion:**

The present study suggested that age, P-wave morphology, LA dimension, failure to graft right coronary artery, and postoperative adrenergic use were independent predictors of post-CABG AF. Therefore, clinical data, ECG and echocardiography may be useful in preoperative risk stratification of the surgical patients for the occurrence of post-CABG AF.

## Introduction

Atrial fibrillation (AF) has been recognized as the most common arrhythmia to occur after coronary artery bypass grafting (CABG). The reported incidence of AF after CABG surgery varies from 20% to 40%, with the arrhythmia usually occurring between second and fourth postoperative days [1-6]. Although this arrhythmia is usually benign and self-limiting, it may result in hemodynamic instability, thromboembolic events, longer hospital stay, and increased healthcare costs [2,4,7,8]. Therefore much attention has focused on the prevention of AF in high risk patients [9]. To find the high risk group, multiple investigations have attempted to identify the demographic risk factors and the predictors of postoperative AF and different results were obtained. These discrepancies may be explained by the differences in the patient profiles, the methods of detection and definition of AF, the small sample size, and the variability in the risk factors evaluated [10]. These studies also included inhomogeneous population of the patients with coronary artery disease, valvular heart disease, and congenital heart disease.

The present study was aimed to identify the preoperative, intraoperative, and postoperative predictors of AF in a pure cohort of the patients with coronary artery disease who underwent CABG surgery.

## Methods

### Study population

Between November 2005 and May 2006, 302 consecutive patients who were scheduled to undergo first on-pump CABG were enrolled in the study. The study was approved by local Ethics Committee, and written informed consents were obtained from all the patients. Previous history of AF or atrial flutter, use of antiarrhythmic drugs other than beta-blockers, uncontrolled heart failure, end-stage renal disease, and presence of an implanted pacemaker were exclusion criteria. Patients were also excluded if they had undergone any surgery other than CABG, if sustained ventricular tachyarrhythmia or cardiogenic shock happened, or if death occurred in operating room. For each patient a form including data related to preoperative, intraoperative and postoperative periods was completed. A standard 12-lead ECG, trans-thoracic echocardiography, laboratory tests and blood pressure measurement were performed in all of the patients.

### Surgical technique

#### 1. On-pump CABG

The patients underwent on-pump CABG by standard surgical technique. Briefly, after a median sternotomy, the ascending aorta was cannulated for the arterial line and a single-stage venous cannula was inserted through the right atrial auricle. Aortic root venting was used and cold crystalloid cardioplegia was administrated through the antegrade route. Cardiopulmonary bypass (CPB) with moderate systemic hypothermia (30-32°C) and moderate hemodilution (Hct >0.22) was used. Intermittent cold crystalloid cardioplegia was administered through the antegrade route in all patients. Peripheral and central anastomoses were constructed during single aortic occlusion. Conduits for bypass included the saphenous veins or internal mammary artery, or a combination of the two.

#### 2. Off-pump CABG

After adequate exposure and stabilization, the target vessel was then exposed and snared above the anastomotic site with a 4-0 prolene suture with a soft plastic snugger to prevent coronary injury. The coronary artery was then opened, and the anastomosis was performed. Visualization was enhanced by the use of a surgical blower-humidifier.

### Postoperative care

After the operation, patients were followed-up in the intensive care unit and were weaned off the ventilator when they fulfilled the following criteria: hemodynamic stability, peripheral temperature >32°C, cooperativity, and no major bleeding. Chest drains were removed on the first postoperative day and the patients were moved to the surgical ward. During the intraoperative period, all patients received unfractionated heparin and protamine sulfate.

All patients were continuously monitored postoperatively during the intensive care unit (ICU) stay. After transfer to the ward, all the patients were connected to monitors for continuous ECG monitoring to the fifth postoperative day. The ward monitor stored the ECG recordings for subsequent analysis. The recordings were analyzed off-line. A12-lead ECG recording was done if necessary to confirm the AF episodes. Two electrophysiologists, who were blinded to other data, reviewed these data on a daily basis. All patients were on beta-blockade medication preoperatively which was continued for entire hospital stay.

The endpoint of study was the occurrence of the new-onset AF during the first week following CABG surgery. AF was defined as absent P wave before the QRS complex together with irregular ventricular rhythm on the rhythm strips. Only AF episodes lasting longer than 5 minutes were counted. Abnormal P-wave morphology, is defined as P-wave duration of more than 110 ms with interpeak notch of more than 40 ms and duration of terminal negative P-wave deflection in lead V1 more than 40 ms.

### Statistical analysis

All continuous variables are presented as mean± SD. Other variables are presented in the percentage of population having a specific value. We tested the association of pre-, intra- and post-operative variables with the occurrence of postoperative AF by using the Student t-test for normally distributed continuous variables and Mann-Whitney U-test for those without normal distribution. Chi-square tests and Fisher's exact probability test (when appropriate) was used for categorical variables. We included all the parameters which showed a P < 0.1 during bivariable correlation to our model of binary logistic regression analysis to determine the independent characteristics associated with postoperative AF. A P-value <0.05 was considered statistically significant. The software SPSS version 13.0 (SPSS Inc., Chicago, IL, USA) was used for statistical analysis.

## Results

### Characteristics of study population

A total of the 302 patients were included in the study. The patient characteristics were summarized in Table 1. Of these patients, 216 (71.5%) were male and 86 (28.5%) were female. The mean age was 58.5±10 years (range, 30 to 79 years) with 96 patients (31%) were ≥ 65 years. On-pump CABG was performed in 282 (93%) and 20 patients (7%) underwent off-pump procedure.

### Characteristics of the patients with AF and those without AF

**Figure 1:**
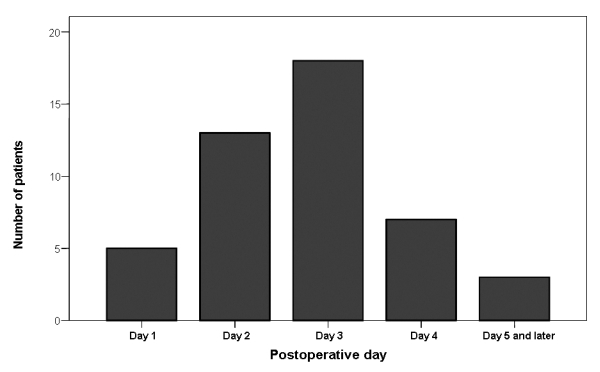
Temporal distribution of the postoperative AF. Note that most of the post-CABG AF occurred between day 2 and day 4.

Of the 302 patients who underwent CABG, 46 patients (15%) developed AF during the study period. The distribution of onset of AF during the postoperative period showed a peak on day 3 (Figure 1) with more than 95 % of episodes occurring before day 5. Patients in the AF group were significantly older than those without AF (63.5±8.5 vs. 57.7±9.9, P< 0.001). Rate of AF was not significantly different in two gender (P=0.7). History of hypertension, diabetes mellitus, chronic heart failure, hypercholesterolemia, hypertriglyceridemia, smoking, prior myocardial infarction as well as the NYHA functional class of patients did not differ significantly between two groups. However, left main coronary artery involvement was significantly higher in patients who developed postoperative AF (P=0.022) (Table 1).

There were also no significant differences in the preoperative and postoperative levels of serum sodium and potassium between the patients with or without postoperative AF (Table 1 and 2). Among the preoperative echocardiography findings (Table 3), LA dimension (38.2±5.4 vs. 34.3±4.8, P<0.001) and severity of mitral regurgitation (P=0.02) were significantly different in the two groups. Although QRS morphology was not significantly different in patients with or without AF, P-wave abnormality (LA abnormality) was seen in 26 % of patients who developed AF and 2 % of patients with no AF (P<0.001). There were no significant differences in the method of surgery, the pump time, and aortic cross-clamp time between two groups (Table2); failure to graft right coronary artery (P=0.002) and postoperative adrenergic use were associated with a higher rate of the postoperative AF (P=0.001) (Table 2).

### Predictors of postoperative AF

By using stepwise logistic regression model, age (OR: 1.067, 95%CI: 1.02-1.116, P=0.005), LA dimension (OR: 1.102, 95%CI: 1.017-1.1936, P=0.017), P-wave morphology (OR: 12.07, 95%CI: 3.35-48.22, P=0.0001), failure  to graft right coronary artery (OR: 3.57, 95%CI: 1.20-10.64, P=0.022), postoperative adrenergic use (OR: 0.35, 95%CI: 0.13-0.93, P=0.036) remained independently predictive of postoperative AF.

## Discussion

AF is the most common complication occurring after cardiac surgery [1-6]. Despite  advances in CPB, cardioplegic arrest, and surgical techniques, its incidence has paradoxically increased in recent years [2] as the result of surgical patients being older and sicker and advances in continuous ECG monitoring technology [3]. It is frequently not well tolerated, and patients may have symptoms including temporary hemodynamic instability, thromboembolic events, and shortness of breath or chest discomfort and has been shown to increase the hospital costs and to lengthen the hospital stay [3,11].

Many preoperative and postoperative factors have been suggested to increase the incidence of postoperative AF after conventional CABG such as advanced age [3], hypertension [3], withdrawal of β-blocker drug [12], right coronary artery stenosis [13], respiratory complications [4], and bleeding [14]. Strategies directed toward reduction of postoperative AF have focused on several drugs, given prophylactically, such as β-adrenoceptor antagonists [11,12], calcium antagonists [15], amiodarone [16], and propafenone [16], with conflicting results. However, little is known about intraoperative mechanisms through which the incidence of postoperative AF could be reduced [17].

The main finding of the present study is that abnormal P-wave morphology is the main independent predictor for the development of postoperative AF, the risk being 12 times higher in the patients with P-wave abnormality compared with those with normal P-wave morphology. This could be related to the fact that abnormal P-wave morphology reflects abnormality of LA size, interatrial conduction defect and LA structural abnormalities. Similar to previous reports [3,4,11,13,18], we also found that age and LA dimension are independent predictors for occurrence of AF after cardiac surgery. However, age and LA dimension were not as powerful as abnormal P-wave morphology.

In the present study, failure to graft right coronary artery was other main predictor of postoperative AF. This finding was supported by the study of Mendes et al [13], who found that presence of the severe right coronary artery stenosis was associated with higher risk of postoperative AF (OR: 3.69, 95% CI: 1.61 to 8.48). We failed to demonstrate the effect of cardiopulmonary bypass on the occurrence of post-cardiac surgery AF. The same result was obtained by Hakala et al [19]. These investigators evaluated the incidence of postoperative AF in 114 patients undergoing on-pump and off-pump CABGs. Despite the similar baseline characteristics, there was no difference in the incidence of postoperative AF in patients undergoing on-pump CABG compared to those with off-pump CABG (36.0% vs. 36.8%, P>0.05).  In another study reported by Ascione et al [20], CPB inclusive of cardioplegic arrest was the main independent predictor of postoperative AF in patients undergoing CABG. These differences in the results of aforementioned studies might be related to the fact that there are important differences in study population, methodology of study, and method of monitoring for the postoperative AF.

Similar to prior reports, we found a significant relation between postoperative AF and postoperative adrenergic use. Salaria et al [21]. investigated the influence of postoperative adrenergic use in 199 patients after cardiac surgery. These investigators showed that adrenergic use was an independent predictor of postoperative AF (OR: 3.35, 95% CI: 1.38-8.12, P=0.016). Drugs with predominantly beta 1-adrenergic receptor affinity were associated with a higher incidence of postoperative AF (dopamine 44%, dobutamine 41%, vs. phenylephrine 20%, P= 0.001).

## Conclusion

The results of the present study demonstrated that P-wave morphology, age, LA dimension, failure to graft right coronary artery, and postoperative adrenergic use were independent predictors of AF after cardiac surgery. Therefore, clinical data, ECG and echocardiography may be useful in preoperative risk stratification of the surgical patients for the occurrence of post-CABG AF. High risk patients detected by these criteria may be proper candidates for the preoperative administration of prophylactic amiodarone or sotalol in addition to standard beta-blockers.

## Figures and Tables

**Table 1:**
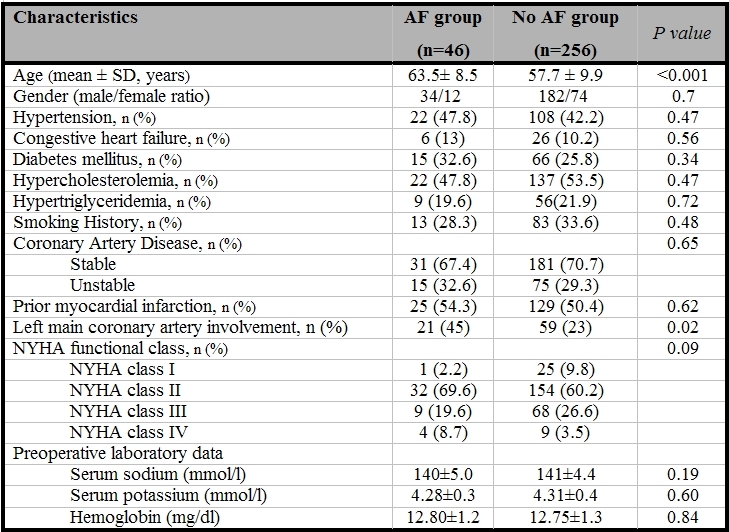
Preoperative clinical and laboratory characteristics of the patients who developed AF and those who did not develop AF after CABG

AF= Atrial fibrillation; NYHA=New York Heart

**Table 2:**
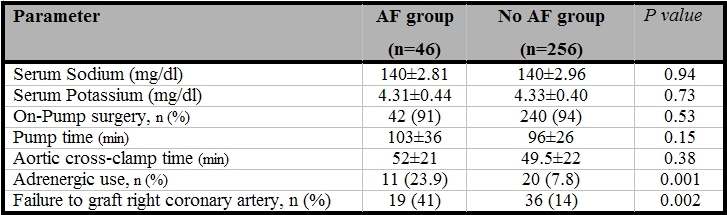
Postoperative characteristics of the patients who developed AF and those who did not develop AF after CABG

AF= Atrial fibrillation

**Table 3:**
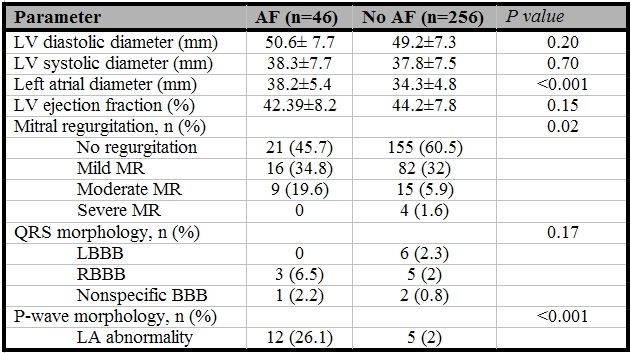
Preoperative echocardiographic and electrocardiographic parameters of the patients who developed AF and those who did not develop AF after CABG

AF= Atrial fibrillation; LV=left ventricle; LA=left atrium; MR=mitral regurgitation; LBBB=left bundle branch block; RBBB=right bundle branch block
